# The complete mitochondrial genome of *Phlugiolopsis damingshanis* (Orthoptera: Tettigoniidae: Meconematinae)

**DOI:** 10.1128/mra.00783-24

**Published:** 2025-05-05

**Authors:** Yuqing Yao, Tinging Yu, Xun Bian, Bin Zhang

**Affiliations:** 1 College of Life Sciences & Technology, Inner Mongolia Normal University 71203 , Hohhot, China; 2 Key Laboratory of Ecology of Rare and Endangered Species and Environmental Protection (Guangxi Normal University), Ministry of Education, Guilin, China; University of California Riverside, Riverside, California, USA

**Keywords:** mitogenome, *Phlugiolopsis*, meconematinae

## Abstract

We present the complete mitochondrial genome of *Phlugiolopsis damingshanis* based on the specimen from Wuming, China. The mitogenome is 16,988 bp in length and is AT rich (70%). It consists of 13 protein-coding, 22 transfer RNA, and 2 ribosomal RNA genes and is identical in gene content to *Phlugiolopsis tuberculata*.

## ANNOUNCEMENT

Meconematinae, commonly known as quiet cricket, produce ultrasonic sound. The genus *Phlugiolopsis* Zeuner, 1940, belongs to the brachypterous group of the tribe Meconematini (1), which was established by Zeuner (2). *Phlugiolopsis* is mainly found in south China (1, 3–6). This region has a subtropical and tropical climate with high humidity, and the soft cuticle of these insects is adapted to such conditions (7). Up to now, the genetic information on species remains limited. *Phlugiolopsis damingshanis* Bian, Shi & Chang, 2012, is a miniature species originally described from specimens from Wuming, Guangxi, China (8). To enrich molecular data and contribute to the phylogeny of *Phlugiolopsis*, the complete mitochondrial genome of *P. damingshanis* was assembled and annotated.

The specimen of *P. damingshanis* analyzed here was collected from Damingshan in Guangxi, China (23.5005 N, 108.436742 W, 1200 m). The voucher specimen is deposited at Guangxi Normal University. The DNA was extracted from the hind femur using the TIANamp Genomic DNA Kit (TIANGEN). The 150 bp paired-end library was constructed with the MGIEasy Kit (MGI) and sequenced on an Illumina NovaSeq 6000 (Illumina Inc.). The raw data were processed using fastp v.0.20.0 (9), by trimming adapters and primers, filtering reads with phred quality <Q5, and filtering reads with N base number >3. The sequencing generated 8,225,010 reads that were filtered. Sequencing was performed by Beijing Berry Genomics Co., Ltd. The sequenced mitogenome data were assembled by NOVOPlasty 4.3.3 (10), yielding an N50 of 16,988 and GC content of 30%. The mitochondrial genome sequence of *Phlugiolopsis tuberculata* (GenBank accession number: NC_068779) was identified as the most closely related reference sequence using BLAST. The start position of the mitochondrial genome was adjusted to correspond with *Phlugiolopsis tuberculata*. The annotation was performed on the MITOS website (11). Gene start and stop positions were confirmed by comparison to seven complete mitochondrial genomes of *Phlugiolopsis* in GenBank and using standard, universally accepted initiation and termination codons as defined in the invertebrate mitochondrial genetic code (12, 13). Nucleotide identities were calculated by BLAST search using the default settings.

**Fig 1:**
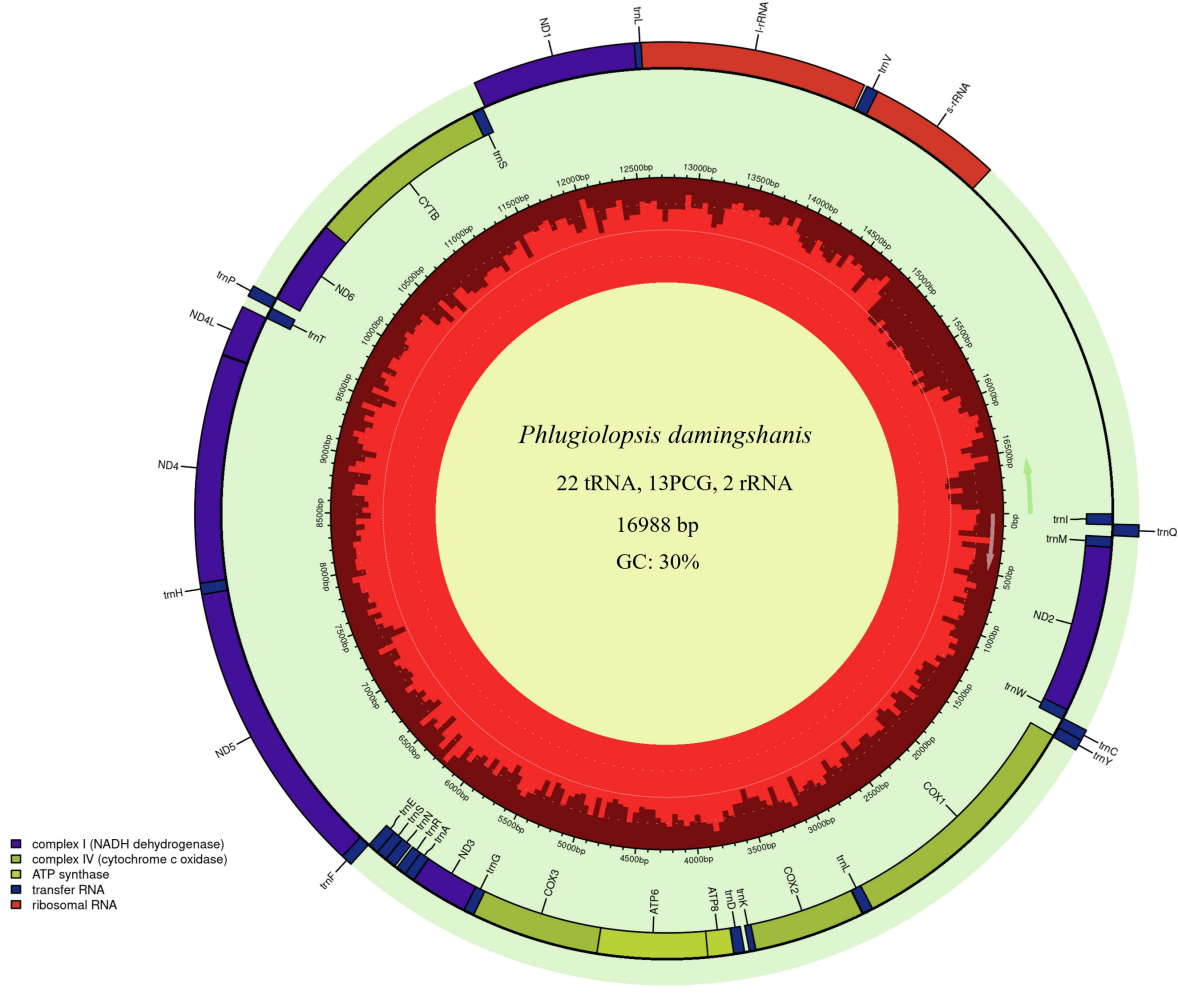
The genome was annotated using the MITOS website and mapped with the Chloroplot online site (16). The innermost ring displays the GC content in red color and the direction of transcription, as indicated by the arrows. The final ring displays the genes. Genes transcribed clockwise are on the inside, while counterclockwise transcriptions are positioned on the outside. The color coding corresponds to genes of different groups as listed in the key in the bottom left. PCG, protein-coding genes.

The complete circular mitochondrial genome of *P. damingshanis* is 16,988 bp in length. The genome contains 37 genes including 13 protein-coding, 22 tRNA, and 2 rRNA (Fig. 1). Six of the protein-coding genes initiate with ATT (ATP8, COX1, ND2, ND3, ND4, and ND5), five with ATG (ATP6, COX2, COX3, ND4L, and CYTB), one with ATA (ND6) and TTG (ND1) (Table 1). Seven of the protein-coding genes terminate with TAA (ATP6, ATP8, COX1, ND2, ND3, ND4L, ND6)，and the T termination codon is found in the rest of five genes except CYTB (TAG) (T stop codon is completed by the addition of 3´ A residues to the mRNA, as is common in animal mitochondrial genomes [14, 15]). The entire mitochondrial genome sequence of *P. damingshanis* is 94.13% similar to the genome of *Phlugiolopsis tuberculata*.

**TABLE 1:** Mitochondrial genome content, organization, and codon information of *Phlugiolopsis damingshanis*

Gene	Type	Minimum nucleotide position	Maximum nucleotide position	Length	Start codon	Stop Codon	Direction
tRNA-Ile	tRNA	1	67	67	–^*a*^	–	Forward
tRNA-Gln	tRNA	64	133	70	–	–	Reverse
tRNA-Met	tRNA	141	205	65	–	–	Forward
ND2	CDS	206	1234	1,029	ATT	TAA	Forward
tRNA-Trp	tRNA	1232	1298	67	–	–	Forward
tRNA-Cys	tRNA	1290	1354	65	–	–	Reverse
tRNA-Tyr	tRNA	1354	1418	65	–	–	Reverse
COX1	CDS	1411	2955	1,545	ATT	TAA	Forward
tRNA-Leu	tRNA	2950	3015	66	–	–	Forward
COX2	CDS	3020	3704	685	ATG	T	Forward
tRNA-Lys	tRNA	3704	3774	71	–	–	Forward
tRNA-Asp	tRNA	3773	3839	67	–	–	Forward
ATP8	CDS	3840	4004	165	ATT	TAA	Forward
ATP6	CDS	3998	4675	678	ATG	TAA	Forward
COX3	CDS	4675	5461	787	ATG	T	Forward
tRNA-Gly	tRNA	5461	5526	66	–	–	Forward
ND3	CDS	5527	5880	354	ATT	TAA	Forward
tRNA-Arg	tRNA	5882	5946	65	–	–	Forward
tRNA-Ala	tRNA	5945	6008	64	–	–	Forward
tRNA-Asn	tRNA	6024	6091	68	–	–	Forward
tRNA-Ser	tRNA	6093	6160	68	–	–	Forward
tRNA-Glu	tRNA	6160	6227	68	–	–	Forward
tRNA-Phe	tRNA	6230	6296	67	–	–	Reverse
ND5	CDS	6297	8028	1732	ATT	T	Reverse
tRNA-His	tRNA	8028	8093	66	–	–	Reverse
ND4	CDS	8094	9420	1,327	ATT	T	Reverse
ND4L	CDS	9426	9722	297	ATG	TAA	Reverse
tRNA-Thr	tRNA	9724	9792	69	–	–	Forward
tRNA-Pro	tRNA	9791	9856	66	–	–	Reverse
ND6	CDS	9858	10385	528	ATA	TAA	Forward
CYTB	CDS	10385	11521	1,137	ATG	TAG	Forward
tRNA-Ser	tRNA	11519	11588	70	–	–	Forward
ND1	CDS	11605	12553	949	TTG	T	Reverse
tRNA-Leu	tRNA	12553	12617	65	–	–	Reverse
16S rRNA	rRNA	12592	13908	1,317	–	–	Reverse
tRNA-Val	tRNA	13923	13994	72	–	–	Reverse
12S rRNA	rRNA	13993	14781	789	–	–	Reverse

^*a*^ –, not applicable.

## Data Availability

The complete mitochondrial genome sequence of *Phlugiolopsis damingshanis* is available in GenBank under accession number PP971631. The associated BioProject, BioSample, and SRA numbers are PRJNA1158430, SAMN43537114, and SRR30593098, respectively. The mitochondrial genome referenced in the text is *Phlugiolopsis tuberculata* GenBank accession number NC_068779.
